# Automatic diagnosis of true proximity between the mandibular canal and the third molar on panoramic radiographs using deep learning

**DOI:** 10.1038/s41598-023-49512-4

**Published:** 2023-12-12

**Authors:** Kug Jin Jeon, Hanseung Choi, Chena Lee, Sang-Sun Han

**Affiliations:** Department of Oral and Maxillofacial Radiology, Yonsei University College of Dentistry, 50-1 Yonsei-ro Seodaemun-gu, Seoul, 03722 Korea

**Keywords:** Dentistry, Diagnosis, Medical imaging, Computer science

## Abstract

Evaluating the mandibular canal proximity is crucial for planning mandibular third molar extractions. Panoramic radiography is commonly used for radiological examinations before third molar extraction but has limitations in assessing the true contact relationship between the third molars and the mandibular canal. Therefore, the true relationship between the mandibular canal and molars can be determined only through additional cone-beam computed tomography (CBCT) imaging. In this study, we aimed to develop an automatic diagnosis method based on a deep learning model that can determine the true proximity between the mandibular canal and third molars using only panoramic radiographs. A total of 901 third molars shown on panoramic radiographs were examined with CBCT imaging to ascertain whether true proximity existed between the mandibular canal and the third molar by two radiologists (450 molars: true contact, 451 molars: true non-contact). Three deep learning models (RetinaNet, YOLOv3, and EfficientDet) were developed, with performance metrics of accuracy, sensitivity, and specificity. EfficientDet showed the highest performance, with an accuracy of 78.65%, sensitivity of 82.02%, and specificity of 75.28%. The proposed deep learning method can be helpful when clinicians must evaluate the proximity of the mandibular canal and a third molar using only panoramic radiographs without CBCT.

## Introduction

The extraction of third molars is the most common surgical procedure performed by dentists and maxillofacial surgeons. Third molar extractions are typically recommended for reasons such as dental caries, periodontal disease, pain, infection, and damage to adjacent teeth^1^. However, the procedure can carry some risks and complications, including swelling, infection, and osteomyelitis. These risks are particularly heightened when a mandibular third molar is located near the mandibular canal, which can result in nerve damage such as nerve paralysis and numbness in the lower lip, gingiva, or jaw^2,3^. The rate of inferior alveolar nerve damage due to third molar extraction has been reported to range from 0.4^4^ to 8.4%^5^, with permanent nerve damage reportedly occurring in fewer than 1% of cases^6,7^. Nerve damage can lead to medico-legal disputes and reduced quality of life for the patient. As such, it is crucial to accurately evaluate the relationship between the tooth and the adjacent mandibular canal prior to performing the surgical procedure.

Panoramic radiographs are used in dental screening to assess various oral issues, as they offer a comprehensive view of the entire oral and maxillofacial region. Moreover, this modality is widely used in dentistry because it involves low radiation exposure compared to other types of radiological images. In a previous study investigating the relationship between the third molar and the inferior alveolar nerve on panoramic radiographs, the accuracy of evaluation of true contact relationships was low, ranging from 52.68 to 69.64%, when assessed by six oral and maxillofacial surgery specialists^8^. Beginners may have lower accuracy and higher variability in diagnostic ability. On panoramic images, the superior and inferior borders of the mandibular canal typically appear as radiopaque lines; however, these borders may be only partially visible or not visible at all^9^. When the mandibular canal is unclear, it can be challenging to locate it or to distinguish it from other structures. Panoramic radiographs are two-dimensional (2D) images, leading to limitations in accurately assessing the positions and relationships of certain structures. Additionally, structures may appear to overlap, making interpretation difficult at times. In particular, molars situated buccal or lingual to the mandibular canal may overlap on panoramic radiographs, causing the appearance of contact between the mandibular canal and the molar. Furthermore, even when the mandibular canal is clearly visible, the true contact relationship should be evaluated using additional cone-beam computed tomography (CBCT) images^10^. However, compared to panoramic radiographs, CBCT involves a higher dose of ionizing radiation, higher costs, and longer imaging times, necessitating caution with pregnant women or young patients. The interpretation of CBCT images also requires specialized training to enable dentists to make the correct diagnosis^11^.

With advances in artificial intelligence technology over the past few years, numerous studies have focused on the automatic detection, classification, and segmentation of various anatomical structures in medical^12^ and dental images using deep learning models^13–15^. In particular, in the dental domain, deep learning has become a popular diagnostic tool for automatically identifying lesions on panoramic radiographs^16,17^. Kwon et al.^16^ developed a deep convolution neural network that automatically diagnosed jaw cysts and tumors with 95.6% accuracy on panoramic radiographs. Ha et al.^17^ reported an artificial intelligence model that automatically detected mesiodens with 96.2% accuracy on panoramic radiographs. Liu et al.^18^ reported an accuracy of 93.3% in evaluating the contact relationship between the mandibular canal and the third molar on CBCT using ResNet-34.

We focused on the principle that objects closer to the imaging plate show distinct images (for example, when evaluating periodontal disease, the lingual bone height is seen more clearly)^9^. It was hypothesized that deep learning could make it possible to diagnose the actual contact relationship between the mandibular canal and the third molar using only panoramic radiographs at the pixel level. In this study, we aimed to develop three deep learning models that enable clinicians to determine the true contact relationship between the third molar and the mandibular canal using only panoramic radiographs, without the need for CBCT images prior to extraction. If the true contact relationship can be automatically diagnosed through artificial intelligence using solely panoramic radiographs, it could provide a second opinion for clinicians in their diagnosis and reduce the risk of nerve damage.

## Materials and methods

### Data preparation

This study was approved by the Institutional Review Board of Yonsei University Health System, Severance Hospital (No. 2-2022-0071), and informed consent was waived due to its retrospective nature. The research was carried out in compliance with pertinent guidelines and ethical regulations, with all data anonymized prior to transmission to the investigators to prevent patient identification. All images were collected from images taken from January 2021 to December 2022, and the image review period was from January to March 2023.

**Figure 1:**
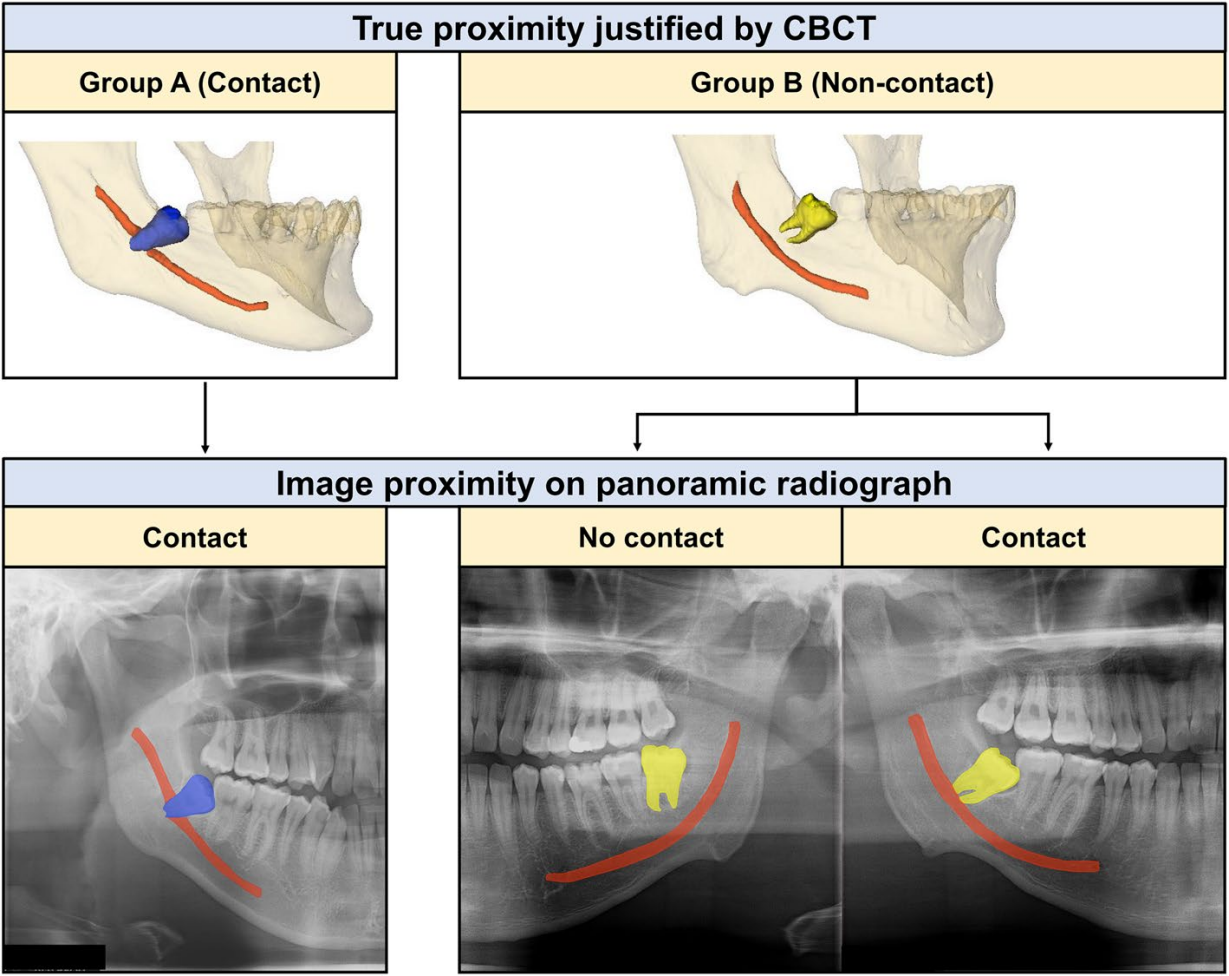
Examples of Group A and Group B on panoramic radiographs.

For algorithm development, panoramic radiograph data were obtained from six instruments at four different institutions. These instruments included Cranex 3^+^ (Soredex Orion Co., Helsinki, Finland), RAYSCAN Alpha (Ray Co., Ltd., Hwaseong-si, Korea), and PaX-i3D (Vatech Co., Ltd., Hwaseong-si, Korea) at Yonsei University Dental Hospital, as well as Osstem-T1-CS (Osstem Co., Ltd., Seoul, Korea), PaX-i3D OP X-ray (Vatech Co., Ltd.), and PHT-35LHS (Vatech Co., Ltd.) at three dental clinics. A total of 901 third molars were assessed from 518 patients who underwent both panoramic radiography and CBCT. All data were classified into Group A (contact) and Group B (non-contact) by two oral radiologists with 25 and 20 years of clinical experience, respectively, according to the true contact relationship between the third molar and the mandibular canal using CBCT images, regardless of the findings on panoramic radiography. Two radiologists evaluated the images after calibration with 10% of the total data (90 third molars) evaluation. In 10% of the total data set, the intra-rater reliability was 0.952 and 0.946. The inter-rater reliability was 0.927. Group A consisted of cases where the third molar and the mandibular canal were in true contact, as evidenced by both panoramic radiographs and CBCT images. Group B consisted of cases in which the third molar and the mandibular canal had no true contact, including both cases in which the third molar and mandibular canal appeared to have no contact and cases where they appeared to be in contact on the panoramic radiographs (Fig. 1). Group A contained 450 third molars and Group B included 451 third molars. The datasets were split into 6:2:2 ratios for training, validation, and test (Table 1).

**Table 1:** Characteristics of the datasets.

Classification	True proximity justified by CBCT	Image proximity on panoramic radiograph	Training	Validation	Test	Total
Group A (contact)	Contact	Contact	270	91	89	450
Group B (non-contact)	Non contact	No contact	124	39	42	205
		contact	147	52	47	246
		Total	541	182	178	901

*CBCT cone beam computed tomography.

### Development of three deep learning models

**Figure 2:**
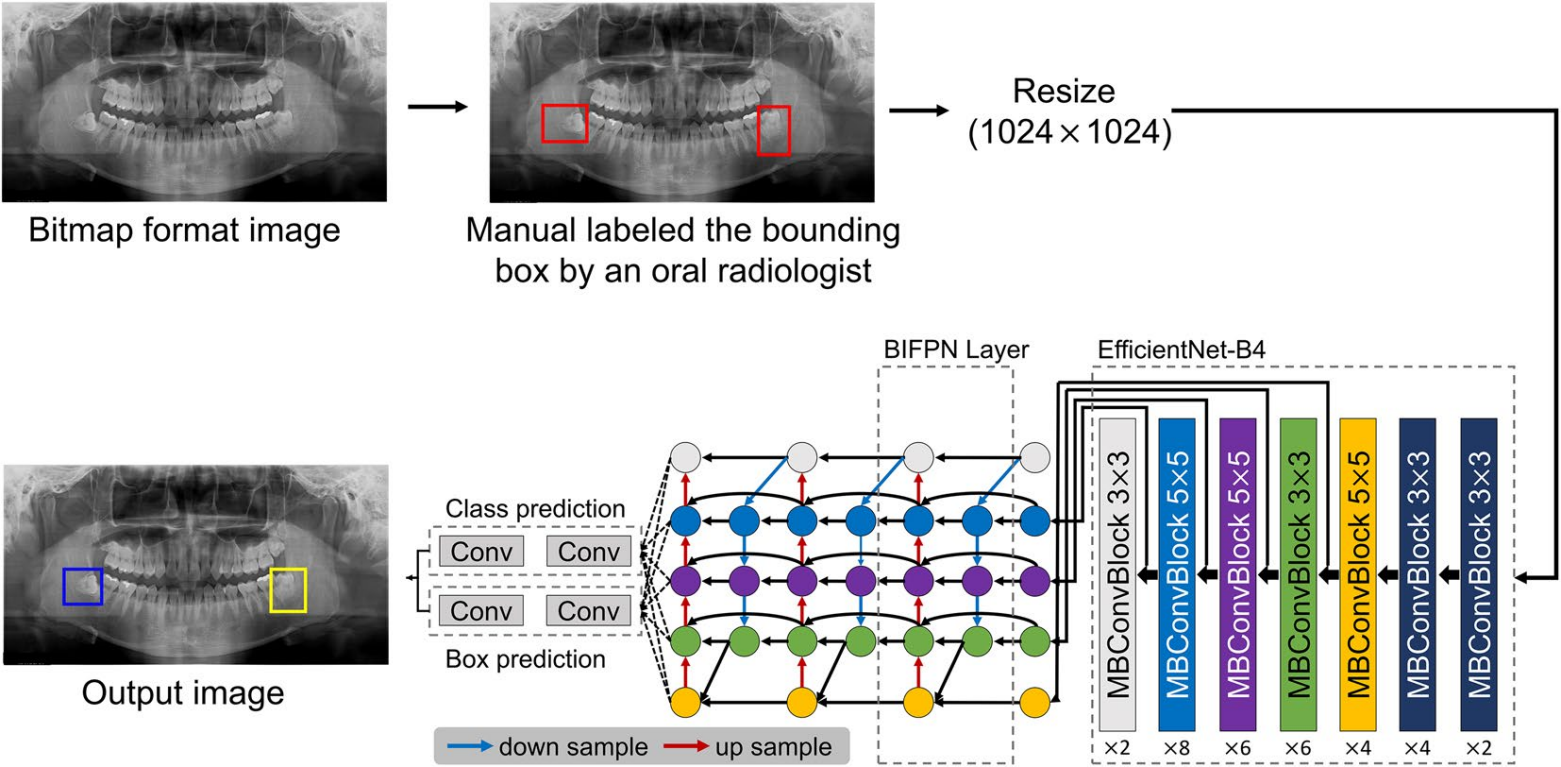
The workflow of the development of a deep learning-based system for predicting the third molar and mandibular canal relationship. Group A (contact) is shown in the blue box, and Group B (non-contact) is shown in the yellow box.

The collected images were in bitmap format, and the size ranged from 1976 to 2988 pixels in width and from 976 to 1500 pixels in height. All images were resized to 1024 (width) and 1024 (height) pixels for model training. An oral radiologist manually labeled the bounding boxes with a size range from 145 to 380 pixels in width and from 200 to 405 pixels in height, which sufficiently included the apex or crown of the third molar and the mandibular canal, using LabelImg software (ver 1.8.4, available at https://github.com/tzutalin/labelImg) as an annotation tool. Additionally, if the mandibular canal was not clearly visible on the panoramic radiographs, it was identified through CBCT images and labeled to include the mandibular canal. These labels served as gold standards for network training. The annotation files contained information about the class names (contact or non-contact) and bounding boxes used to describe the location of the third molar and mandibular canal. Figure 2 shows the overall process of developing a deep learning-based diagnostic system for the third molar and mandibular canal relationship.

**Figure 3:**
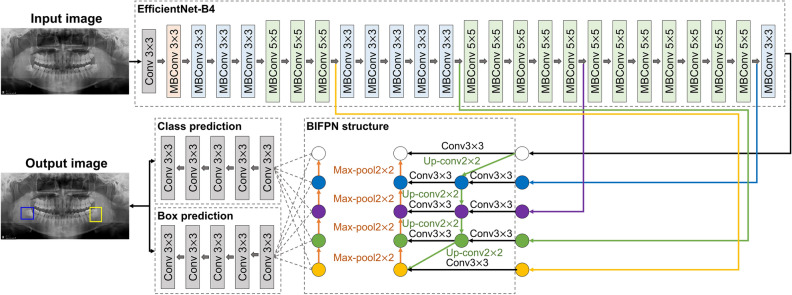
The EfficientDet-D4 architecture for automatic diagnosis of third molar and mandibular canal access on panoramic radiographs. Group A (contact) is shown in the blue box, and Group B (non-contact) is shown in the yellow box.

The detection and classification model of the relationship between the third molar and mandibular canal was developed by using three deep learning algorithms (RetinaNet, YOLOv3, and EfficientDet) on panoramic radiographs. RetinaNet^19^ is designed to detect features of various sizes in images by employing ResNet50 and the feature pyramid network (FPN) model, a multi-scale feature pyramid, as its backbone. The feature maps for each pyramid level extracted from the backbone are input into the classification subnetwork and bounding box regression subnetwork, respectively, to detect the target. This model was the first for which the use of a focal loss function, focused on class imbalance problems to improve object detection performance, was proposed. In YOLOv3^20^, darknet 53 is used as the backbone and binary cross-entropy as the loss function, demonstrating rapid inference time and robust performance. This algorithm is widely used in medical imaging. It was trained to automatically detect the relationship between the third molar and mandibular canal on panoramic radiographs by using 53 convolutional layers and the leaky rectified linear unit (ReLU) activation function, which generated three feature maps with different resolutions. EfficientDet^21^ employs EfficientNet-B0 to B6 as the backbone, focusing on improving accuracy and efficiency with fewer parameters than previous detectors. In the present study, the EfficientDet-D4 model was used due to computer resource limitations. Similar to RetinaNet, this model uses a focal loss function but employs the bidirectional FPN (BiFPN) model to detect objects of various sizes when extracting features from images. Unlike the conventional top-down pathway FPN, a bottom-up pathway was added for bidirectional use, and weights for different features were considered by connecting input and output nodes. Behind the BiFPN layer, a classification sub-network and a box prediction subnetwork are used to detect objects (Fig. 3).

All models were trained using transfer learning on the COCO dataset^22^ for 300 epochs with a batch size of 2 and momentum of 0.9. During training, predicted bounding boxes were considered correct if the detected region had an intersection-over-union value of 0.5 or greater compared to the annotation. During testing, when the trained model detected the third molar and mandibular canal, the detected area on the panoramic radiograph was marked with a box and class name. Group A (contact) is shown in blue boxes, while Group B (non-contact) is shown in yellow boxes. If no third molar was detected, the input image was returned without a box. All networks were implemented in Python 3 using the Keras library with a TensorFlow backend and trained on an NVIDIA Titan RTX graphics card (NVIDIA, Santa Clara, CA, USA).

### Evaluation of the three deep learning models

The three models successfully detected the third molars and mandibular canals for all test data sets. Consequently, the performance of these models was evaluated using the classification performance indicators of accuracy, sensitivity, and specificity. The accuracy, sensitivity, and specificity were determined based on the classification of the relationship between the third molar and the mandibular canal, using the following equations:

1 $${\text{Accuracy}} = \frac{{{\text{TP }} + {\text{ TN}}}}{{{\text{TP }} + {\text{ TN }} + {\text{ FP }} + {\text{ FN}}}}$$

2 $${\text{Sensitivity}} = \frac{{{\text{TP}}}}{{{\text{TP }} + {\text{ FN}}}}$$

3 $${\text{Specificity}} = \frac{{{\text{TN}}}}{{{\text{TN }} + {\text{ FP}}}}$$

where TP, TN, FP, and FN represent true positive, true negative, false positive, and false negative, respectively.

## Results

**Figure 4:**
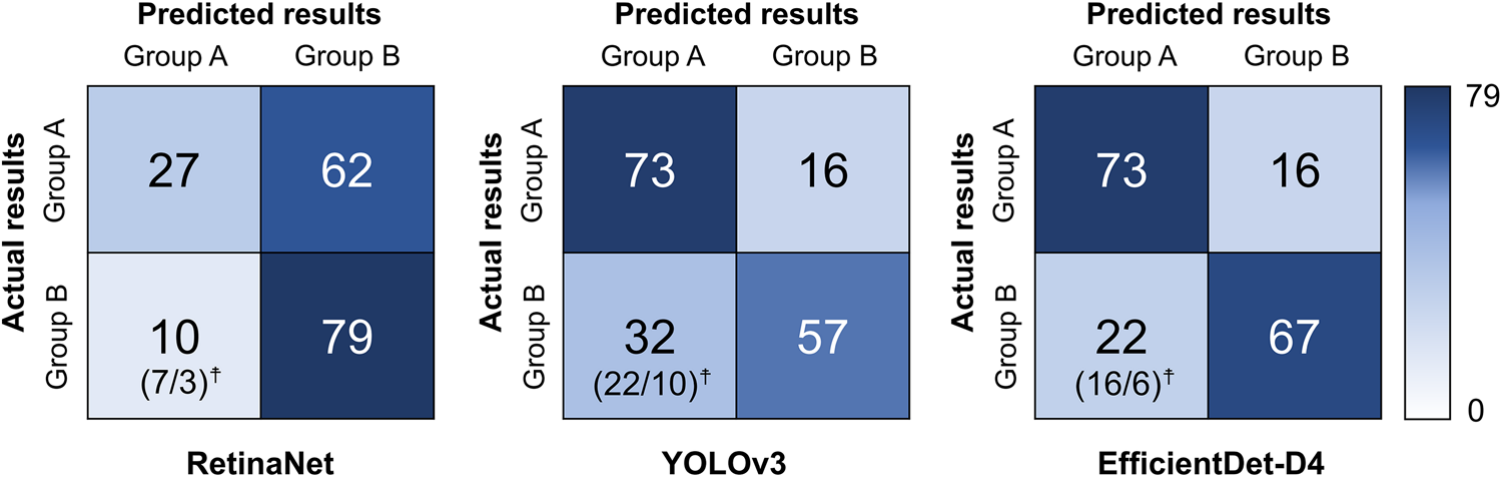
Confusion metrics of the automated deep learning-based classification models on the test datasets. ( )^☨^ indicates the number of contact and non-contact instances on panoramic radiographs, respectively, for Group B (non-contact) predicted as Group A (contact).

**Figure 5:**
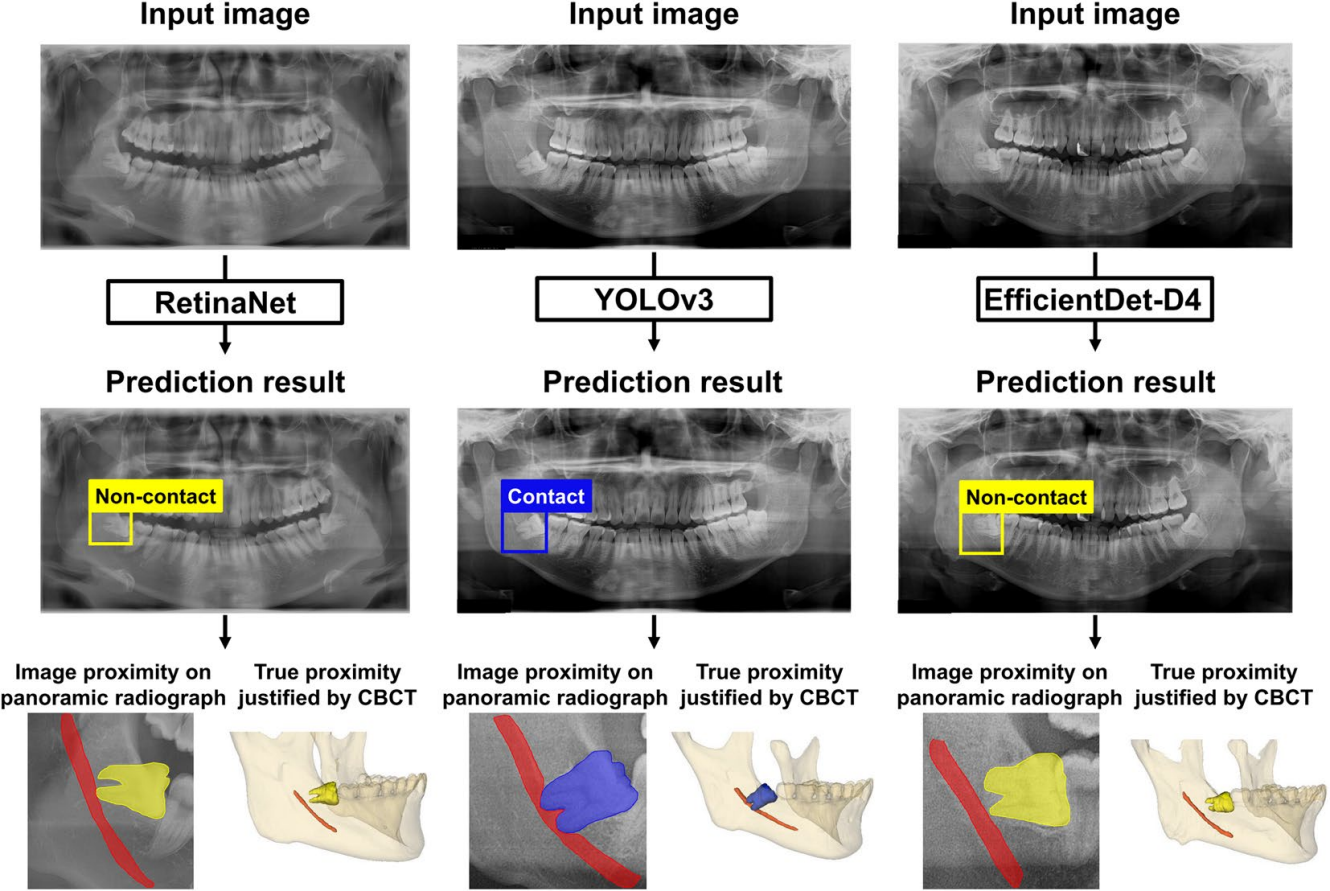
Examples of correct classification of the relationship between the third molar and the mandibular canal in three models.

Table 2 illustrates the diagnostic performance of the three implemented models in relation to the association between the third molar and the mandibular canal. EfficientDet-D4 showed an accuracy of 78.65%, a sensitivity of 82.02%, and a specificity of 75.28%. YOLOv3 showed an accuracy of 73.03%, a sensitivity of 82.02%, and a specificity of 64.04%, and RetinaNet showed the highest specificity (88.76%). RetinaNet, YOLOv3, and EfficientDet-D4 incorrectly predicted Group A (contact) samples as Group B (non-contact) at rates of 69.66%, 17.98%, and 17.98%, respectively. In contrast, the rates of incorrectly predicting Group B (non-contact) samples as Group A (contact) for RetinaNet, YOLOv3, and EfficientDet-D4 were 11.24%, 35.96%, and 24.72%, respectively, with a higher number of contact cases observed on the panoramic radiographs (Fig. 4). Figure 5 displays correctly predicted examples from the three models using the test dataset.

**Table 2:** Diagnostic performances of the three models implemented.

	Accuracy (%)	Sensitivity (%)	Specificity (%)
RetinaNet	59.55	30.34	88.76
YOLOv3	73.03	82.02	64.04
EfficientDet-D4	78.65	82.02	75.28

## Discussion

We developed three deep learning models to determine the true contact relationship between the third molar and the mandibular canal using only panoramic radiographs, with the gold standard being two radiologists’ evaluations of the contact relationship from CBCT. Compared to specialists and deep learning in previous studies, our study demonstrated superior accuracy in automatically diagnosing the true proximity of the third molar and mandibular canal on panoramic radiographs. Conducted using images from various devices across multiple institutions, this study is believed to be highly applicable in clinical practice. Dentists perform extractions several times a day, and extracting mandibular third molars is a challenging task due to their proximity to the mandibular canal. Before extraction, the closeness of the mandibular canal and a third molar is primarily assessed through panoramic radiographs. Identifying the mandibular canal on these images can be difficult, and due to the limitations of 2D imaging, two structures separated by the buccal and lingual sides may appear to overlap and make contact. Diagnosing true proximity on panoramic radiographs without CBCT is extremely challenging, even for specialists.

Several studies have recently attempted to automatically diagnose the contact relationship between third molars and the mandibular canal using deep learning on panoramic radiographs^8,23,24^. Our study is particularly robust, as we collected data from six devices across multiple institutions and included all possible cases to improve real-world usability. Furthermore, our models were developed to diagnose true contact on original panoramic radiographs rather than cropped images, resulting in improved accuracy compared to previous studies. Fukuda et al.^23^ conducted an experiment in which they cropped 600 panoramic radiographs to 70 $\times$ 70 and 140 $\times$ 140 pixels, and they classified contact and non-contact groups of the third molar and mandibular canal on images using AlexNet, GoogLeNet, and VGG16. The diagnostic performance was highest, at 0.92, after training with 70 $\times$ 70 pixels on GoogLeNet. Although the accuracy was as high as 0.92, which exceeded that of the present study, that experiment was performed using panoramic radiographs cropped to include the apex of the third molar and the mandibular canal as input images. This approach has a major drawback in that it is unknown whether the third molar truly comes into contact with the mandibular canal. Choi et al.^8^ proposed the ResNet50 model to determine the true contact relationship between the third molar and the mandibular canal using 571 panoramic radiographs and compared the performance of the artificial intelligence model to that of oral and maxillofacial specialists. In that study, true contact analysis was classified using only cases in which the third molar and mandibular canal overlapped on images. The accuracy of the deep learning model was 0.63 for true contact and 0.76 for the buccolingual position (non-contact), constituting better performance than six specialists (52.68–69.64% and 32.26–51.61%, respectively). However, the mandibular canal may not be identified on such images, and some clinicians may not accurately evaluate the contact between the third molar and the mandibular canal on an image. Sukegawa et al.^24^ reported a deep learning model using ResNet50 and RestNet50v2, which was developed to classify the contact state between the mandibular canal and the third molar. The dataset included 1279 panoramic radiographs cropped to 250 $\times$ 200 pixels. Two experiments were conducted: contact analysis on panoramic image and true contact analysis between the third molar and the mandibular canal. In both sets of experimental results, the ResNet50v2 model showed the best performance, with accuracies of 0.860 and 0.766, respectively. The true contact analysis was similar to our study but differed in that it involved manually cropped images. In our study, the entire image was used without manual cropping, and RetinaNet, YOLOv3, and EfficientDet-D4, which differ from the models used in previous studies, were employed. Among the three models, EfficientDet demonstrated the highest accuracy at 78.65%, which exceeded the result of the true contact analysis by Sukegawa et al. (76.6%).

In the present study, EfficientDet-D4 demonstrated the highest accuracy, at 78.65%, for determining the relationship between the third molar and the mandibular canal, while YOLOv3 and RetinaNet had accuracies of 73.03% and 59.55%, respectively. Among the three models, RetinaNet had the lowest accuracy (59.55%), but it exhibited the highest specificity at 88.76%. Overall, we observed that the sensitivity (i.e., correctly predicting true contact) was higher than the specificity (i.e., correctly predicting non-contact) on panoramic radiographs. Cases where actual non-contact is incorrectly predicted as contact are not overly problematic from a practical standpoint, as the clinician can be particularly careful when extracting the third molar, but predicting contact as non-contact may increase the risk of nerve damage. Therefore, high sensitivity is more important than high specificity.

In cases where CBCT access is limited, the model developed in this study can provide a second opinion about the actual proximity of the third molar and the mandibular canal using only panoramic radiographs.

The present study had several strengths. First, we fully automated the analysis of the true contact relationship between the third molar and the mandibular canal using only panoramic radiographs and a deep learning model. Unlike previous studies utilizing cropped images, our algorithm was developed with original panoramic radiographs, accounting for the real clinical environment. Second, we developed models reflecting the characteristics of real-world data by using images acquired from various devices across multiple institutions. This showcases the potential for application in the dental field. However, the accuracy of the current model reaches only up to 78.65%, indicating that further efforts are necessary to enhance the accuracy above 80% by adjusting parameters and investigating other deep learning models.

## Conclusion

Various deep learning models have been developed to automatically identify the true contact relationship between the third molar and the mandibular canal on panoramic radiographs. By utilizing the automatic true proximity analysis model developed here, it is feasible to determine the true contact relationship between the third molar and the mandibular canal using only panoramic radiographs, without the need for CBCT images. This algorithm can offer diagnostic assistance to inexperienced dentists.

## Data Availability

The data generated and analyzed in this study are not publicly available due to Korean privacy laws and policies, but they are available from the corresponding author upon reasonable request.
